# Sex Differences in Asthma: A Key Role of Androgen-Signaling in Group 2 Innate Lymphoid Cells

**DOI:** 10.3389/fimmu.2017.01069

**Published:** 2017-08-31

**Authors:** Sophie Laffont, Eve Blanquart, Jean-Charles Guéry

**Affiliations:** ^1^Centre de Physiopathologie de Toulouse Purpan (CPTP), Université de Toulouse, INSERM, CNRS, UPS, Toulouse, France

**Keywords:** sex differences, group 2 innate lymphoid cells, allergic asthma, sex steroid hormones, androgens, androgen receptors

## Abstract

Infectious diseases, autoimmune diseases, and also allergy differentially affect women and men. In general, women develop strongest immune responses and thus the proportion of infected individuals and the severity of many viral, bacterial, or parasitic infections are increased in men. However, heightened immunity in women makes them more susceptible than men to autoimmunity and allergy. While sex differences in immunity are well documented, little is known about the cellular and molecular mechanisms underlying these immunological differences, particularly in allergic asthma. Asthma is a chronic inflammation of the airways mediated by exacerbated type 2 immune responses. Sex differences have been reported in the incidence, prevalence, and severity of asthma. While during childhood, males are more susceptible to asthma than females, there is a switch at the onset of puberty as for many other allergic diseases. This decrease of asthma incidence around puberty in males suggests that hormonal mediators could play a protective role in the susceptibility to allergic responses in male. Group 2 innate lymphoid cells (ILC2s) have recently emerged as critical players in the initiation of allergic responses, but also in the resolution of parasitic infection, through their capacity to rapidly and potently produce type 2 cytokines. This review will cover the current understanding of the impact of sex-linked factors in allergic inflammation, with a particular focus on the role of sex hormones on the development and function of tissue-resident ILC2s.

## Introduction

Sex differences in asthma prevalence and phenotypes have been well described (1–3). Whether such sex bias in asthma incidence and severity relies mostly on inherent differences between sexes in lung structure, anatomy, or physiology, which could be influenced or not by sex steroid hormones (4), or differences in immunity between sexes due to the direct action of sex steroid hormones on particular immune cell populations (5, 6), or both, is not clearly understood. In this review, we discuss the research on the role of sex hormones in the immunoregulation of allergic asthma. We summarize recent findings highlighting the protective action of male sex hormone androgens, through the negative regulation of group 2 innate lymphoid cells (ILC2s).

## Immunological Mechanisms of Allergic Asthma

Asthma is a prototypical type 2 immune response-mediated disease triggering chronic inflammation of the airways (7). As for many allergic diseases, asthma incidence, prevalence, and severity are different between men and women (1–3). Allergic asthma due to sensitization to environmental allergens is associated with type 2 inflammations in the majority of children and adult patients (3). Allergic lung inflammation is primarily driven by the over production of type 2 cytokines, IL-4, IL-5, and IL-13, in response to inhaled allergens, such as those derived from house dust mites, pets, and pollens. IL-4 produced by Th2 cells is critical for allergen-specific IgE production, which is responsible for the release of inflammatory mediators upon cross-linking of the high affinity IgE receptors on the surface of mast cells and basophils (7). IL-5 is responsible for the recruitment of eosinophils, which together with IgE and mast cell responses are characteristic of allergic asthma. Many of the asthma-related allergens exhibit enzymatic activity that damage the epithelial cell (EC) layers and enable them to activate mucosal dendritic cells (DCs), which then migrate to draining lymph nodes where they activate allergen-specific naive CD4 T cells and promote their differentiation into type-2 cytokine-producing Th2 cells (7). Although Th2 cells have long been thought to be the crucial and unique source of type-2 cytokines in asthma, the recent discovery of ILC2 revealed a new reservoir of type-2 cytokines in the lung (8). ILC2s are potent source of IL-13 and IL-5. They also produce IL-4, although at lower level compared to Th2 cells. ILC2 activation promotes eosinophil infiltration, mucus secretion, and airway hyperreactivity, but not IgE production, which requires the development of adaptive Th2 responses (9). Recent evidence has emphasized the critical role of ILC2-derived type 2 cytokines in allergen-induced Th2-dependent lung inflammation. ILC2s depend on both the transcription factors GATA-3 and RORα for their development (10, 11). GATA-3 is essential for the development of ILC2 progenitors (ILC2p) in the bone marrow and for maintaining the mature ILC2 population in the periphery (10). GATA-3 is therefore required for the cell fate determination of both ILC2 lineage and Th2 cells. Besides GATA-3, RORα, a member of the retinoic acid related orphan receptor family, which plays a partially redundant role in differentiation of the Th17 cells (12), has been shown to be critical for the development and function of ILC2 (11). Staggered sg/sg mice with a deletion in the RORα ligand binding domain exhibit a drastic reduction in the number of ILC2 in periphery and are unable to mount an efficient innate response to intestinal parasites (11). This mouse model of ILC2-deficiency has been used to demonstrate the critical role of ILC2 in the initiation of Th2 responses in the lung to the allergen protease papain (13). ILC2-derived IL-13 was shown to promote the migration of lung DCs into the draining lymph node where they initiate Th2 cell differentiation (13). The production of IL-33 by ECs was critical for allergen-induced ILC2 activation and type 2 lung inflammation in this model (13). Besides IL-33, ILC2s sense other epithelial derived cytokines, such IL-25 and TSLP, and also multiple environmental cues such as mast cells derived eicosanoids or lipid mediators like leukotriene D4 (LTD4), prostangladin D2, or lipoxin A4 (14–18). LTD4 and PGD2 act in synergistic manner with airway epithelial cytokines to promote IL-13 production by ILC2s (14, 15, 17). Whereas lipoxin A4, a natural resolving mediator which expression is reduced in severe asthma, appears as a unique negative regulator of IL-13 release by human ILC2s (18).

Investigating the ILC2-requirement for allergic lung inflammation development by multiple sensitizations with HDM as compared to the classical model of active immunization with ovalbumin (OVA) revealed interesting distinctions. Whereas ILC2 were required in the HDM-induced asthma model, they were largely dispensable in the OVA model in which Th2 cells are first primed by intraperitoneal injection of OVA in alum (19). Thus, ILC2s appear critical during the priming of adaptive Th2 response to inhaled allergens, whereas they seem dispensable for the stimulation of memory Th2 cells (19).

**Figure 1:**
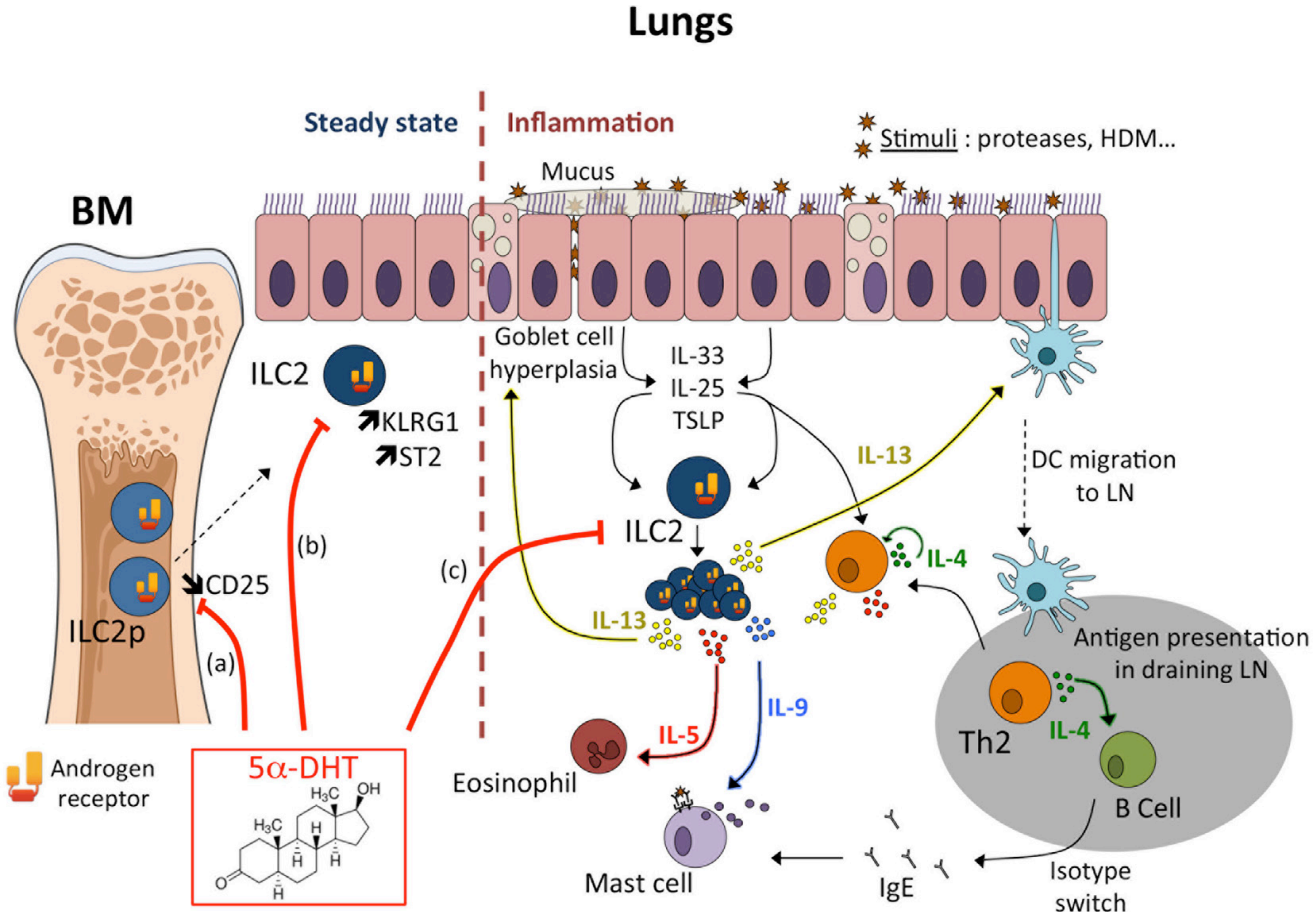
Mechanisms of androgen-mediated inhibition of type 2 inflammation. Androgens mediate the decreased recruitment/proliferation of group 2 innate lymphoid cell (ILC2) responsible for the reduced development of type 2 inflammation in males compared to females. Androgen-signaling may act at multiple levels to limit ILC2 development at steady state and/or ILC2 response during inflammation. (a) At steady state, androgen (5α-DHT) would limit the development of ILC2 progenitors (ILC2p) in the bone marrow (51). Whether this may negatively impact the seeding of ILC2 in peripheral tissues is unknown, as well as the period of life where this mechanism may occur. Enhanced CD25 expression in female ILC2p correlated with an increased frequency of proliferating (Ki67^+^) cells (51), suggesting that greater sensitivity to IL-2 in female ILC2p could promote ILC2 seeding in non-lymphoid tissues during ontogeny. This hypothesis is however not supported by recent findings demonstrating that CD25-deficient tissue-resident ILC2 proliferated to a similar extent as WT ILC2 in bone marrow chimeric mice, both at steady state and during acute helminth infection (54). Moreover, few ILC2s develop from bone marrow progenitors in adult mice (54). However, this hypothesis has recently been called into question by the characterization in humans of progenitors common to all ILCSs (ILCPs) in blood and tissues, suggesting that differentiation of ILCs could occur on demand in tissues and at any age (55). (b) Tissue-resident ILC2 numbers are reduced in the lungs of male mice and exhibit enhanced expression of ST2 and KLRG1 (51). This observation is counter intuitive as it was associated with reduced functional properties of tissue-resident male ILC2s (50). *In vivo* and *in vitro* KLRG1 expression is robustly controlled by androgen receptor-signaling (51). (c) ILC2s play an important role during allergic airway inflammation. Allergens or proteases trigger the production of the alarmins (TSLP, IL-33, IL-25) by epithelial cells (ECs). EC-derived cytokines act on tissue-resident ILC2 that produce IL-13, a key cytokine for Th2-mediated inflammation. Most of the sex differences observed in the IL-33-driven inflammation model could be already imprinted by the preexisting sex bias in tissue-resident ILC2 reflected in the steady-state numbers (51); however, we cannot rule out a direct effect of androgen-signaling at the time of ILC2 expansion *in vivo*.

The discovery of ILC2 has shed new lights on the understanding on how inhaled allergens induce type 2 lung inflammation. A common pathway of induction of allergic airway inflammation is emerging in which allergens provoke the release of alarmins, such as IL-33, which stimulate lung-resident ILC2 to produce type 2 cytokines able to cause inflammation associated with allergic disease and to promote Th2 cell activation and the subsequent development of adaptive B cell responses and IgE production (Figure 1) (9).

### Sex Differences in Allergic Asthma

Asthma is not constant and can vary across the life course of the patient, who can experience period of remission and new asthma onset (3). This is particularly true if one considers asthmatic patients with onset during childhood. Whereas the prevalence of asthma is higher in males than in females before adolescence, the onset of puberty reverses that trend not only for asthma but also for most allergic disorders (1–3) (Box 1). Although this switch was attributed to a late incidence of asthma among girls (2, 20), a more recent study reported a higher rate of asthma remission in boys than in girls (21). During the intervening years between the ages of 10 and 18, the percentage of children who grew out of asthma is 39.4% in boys and 23.4% in girls (21). Again, acquisition of asthma during this time period tended to be higher in girls than in boys. As a consequence, sex bias in asthma prevalence during childhood reverses in adolescence and in young adults (1–3). Thus, the drop in asthma incidence observed in and around the time of puberty in males, as well as the increased numbers of remission observed in males is strongly suggestive of a protective action of male sex hormones (1, 2). Besides asthma, the protective role of puberty has also been reported in other allergic diseases, such as vernal keratoconjunctivitis, a severe form of ocular allergy, mainly occurring in boys, which almost disappears after puberty (22) (Box 1). These observations fit with the generally accepted model that androgens, which are produced at higher concentrations in postpubertal men, generally suppress immune cell reactivity (23). This immunosuppressive effect of androgens may reflect the inhibitory action of androgen receptor (AR) signaling on a critical subset of immune cells. However, despite the observations that testosterone inhibits immunity in a variety of systems, the precise molecular mechanisms by which androgens achieve this effect are still poorly understood (23, 24). Recently, androgen-signaling was shown to alter Th1 cell immunity through the inhibition of Il-12-Stat-4 signaling in CD4 T cells (24). This is a rare example of a well-documented inhibitory effect of androgen on T-cell immunity; however, this effect should promote rather than suppress Th2-mediated allergic responses, and certainly cannot account for a possible immunosuppressive action of androgen on the development of type 2 lung inflammation.

Box 1 **Allergic disease negatively regulated by puberty in male.**

Besides allergic asthma, recent studies have also indicated a change in gender prevalence for allergic disorders such as rhinitis (25), food allergy (26), eczema (27), and vernal keratoconjunctivitis (VKC) (22). Reports on rhinitis have shown that gender and atopy are two factors influencing the natural history of the disease. Whereas atopic rhinitis increases in both sexes from 1 to 18 years, without major changes during puberty, non-atopic rhinitis decreases in prevalence during adolescence, resulting in female dominance at 18 years (25). Analysis of sex disparity in food allergy among the literature for IgE-mediated allergy to 11 allergenic foods indicated a switch in female sex ratio from male predominance in childhood (male/female ratio, 1.80) to female dominance in adulthood (male/female ratio, 0.53) (26). Epidemiological studies have shown that eczema is more prevalent among boys during childhood with girls being more frequently affected after puberty (28, 29). Trends in the prevalence of eczema in the course of childhood and adolescence have been examined longitudinally in a prospective cohort study from 1 to 18 years (27). During adolescence, girls develop eczema more frequently than boys, suggesting a gender reversal during adolescence (27). Taken together, these observations indicate that while male predominance may be seen in early childhood for many allergic diseases, a switch to female predominance occurs by early adulthood. VKC is another interesting example of gender-specific protective action of pubertal factors. VKC is a severe form of ocular allergic conjunctivitis mainly occurring in children and young adults. Although the disease is rare in Europe 3.2/10000, it is almost endemic in subtropical countries (22, 30). Hyper IgE is found in 50% of the patients and VKC has been related to T cell-mediated responses, massive eosinophil infiltration, and can be triggered by non-specific hyper-activity (sunlight, wind, dust). VKC typically occurs between the age of 4 and 12 years, more frequently in boys, with male/female sex ratio ranging from 3/1 to 5/1. The disease spontaneously disappears after puberty in 90% of patients. With the notable exception of atopic rhinitis, all these observations are supportive for a protective action of puberty in male.

### Sex Hormones in Type 2-Mediated Allergic Airway Inflammation

Using the OVA model of allergic asthma where Th2 cells are first primed by intraperitoneal injection of OVA in alum, followed by intranasal challenge later on, sex differences in the development of airway inflammation have repeatedly been reported. In most studies, lung eosinophil infiltrates, serum IgE production, and type-2 cytokine production in lung tissues were higher in female than in male (31–33). This was associated with enhanced airway hyperresponsiveness and airway remodeling (31–33). Enhanced susceptibility of female to all cardinal features of allergic asthma was also observed in another model induced by intranasal exposure to HDM (32). Interestingly, in an earlier study, castration of male mice abolished the difference with female, suggesting a negative regulatory role of androgens (34). It has also been reported that administration of dehydroepiandrosterone (DHEA), a natural steroid hormone secreted by the adrenal gland and converted into androgens or estrogens, suppresses eosinophil infiltration and airway hyperresponsiveness in the OVA-induced asthma model, although the mechanisms are still not known (35). Females ovariectomized before sensitization to OVA had reduced production of IL-5 and lower numbers of eosinophils in bronchoalveolar lavage (BAL) fluid (36). However, the role of estrogens in this deleterious effect was not established. Actually, estradiol administration in castrated female mice during the effector phase of the response to inhaled antigen was found to inhibit lung eosinophilia (36), in agreement with works by others in experimental model of peritonitis (37). Thus, the effects of estrogens in asthma appear contradictory and complex, probably due to the pleiotropic expression of the estrogen receptors (ERs) in various tissues, beside immune cells. Estrogens mediate both transcriptional and non-genomic effects through either ER α or β. In the absence of estrogens, growth factor signaling can induce ER phosphorylation and activation in a ligand-independent manner (38). Both ERs are expressed in the lung, particularly ERβ (39). ERα-deficient female mice exhibit spontaneous airway hyperresponsiveness under basal conditions (40). ERα-deficiency had limited impact on airway inflammation after allergen challenge, and no differences were observed between WT and ERα^−/−^ mice in BAL fluid levels of eosinophils, IL-4 and IL-5 (40). However, this exacerbating effect of ERα deficiency on airway hyperreactivity at steady state and upon antigen sensitization in female mice suggested that estrogen signaling acted as a negative regulator of airway hyperresponsiveness in mice. Nevertheless, castration or estrogen-supplementation of WT mice failed to recapitulate the phenotype of constitutive ERα-deficient mice, suggesting a ligand-independent mechanism of action. ERα-deficiency, however, did not alter the inflammatory response in the airway, suggesting that ERα was acting at the level of airway smooth muscle and nerves, rather than in immunocompetent cells (40). Thus, while these results indicate that ERα expression can negatively regulate airway hyperreactivity, they cannot explain the enhanced susceptibility of female to allergic asthma. Although we cannot exclude that estrogens might signal in tissue-resident immune cell subsets such as DCs (41) or macrophages (42) to regulate type 2 inflammation, direct evidence for a role of estrogen as a critical mediator of the sex differences in asthma are still lacking.

## Evidence for a Protective Role for Androgens in the Susceptibility to Type 2 Immunity

Few studies have documented a negative regulatory role of androgens or their precursors on the development of airway inflammation associated with asthma. As mentioned earlier, DHEA administration inhibited eosinophil infiltration and airway hyperresponsiveness in the OVA-induced asthma model (35). In another model of asthma using the dust mite *Dermatophagoides farinae*, DHEA intake inhibited airway inflammation (43). In human clinical trial, intranasal administration of DHEA-3-sulfate (DHEAS) improved asthma in subjects with poorly controlled moderate-to-severe asthma (44). However, the *in vivo* mechanisms of actions of DHEA are complex and could be mediated through multiple signaling pathways involving specific membrane receptors. Alternatively, although no unique DHEA or DHEAS nuclear steroid receptor has been found, DHEA and DHEAS can be transformed into more potent sex steroids able to activate both ARs or ERs. *In vivo*, DHEA can be converted into androstendione and testosterone, which is the direct precursor of both 17β-estradiol and 5α-DHT (Dihydrotestosterone), through the respective contribution of aromatase or 5α-reductase enzymes (45).

Sex-related differences in the susceptibility to parasitic infections associated with type 2 immune responses have also been reported. In most cases, females were superior to males in their ability to clear infection by helminths such as *Nippostrongylus brasiliensis* (46). In line with this hypothesis, work carried out in the 1990s in a rodent species (*Millardia meltada*) revealed a greater susceptibility of males to infection with *N. brasiliensis*. Androgens were responsible for this difference in susceptibility to infections, indeed orchidectomy was associated with a better ability to fight infection, whereas castration of females had no effect. Finally, testosterone administration in females inhibited expulsion of the parasite and was associated with decreased number of goblet cells in the intestinal mucosa (47, 48). Given the central role of ILC2 in the innate response to intestinal parasites (49), it is tempting to speculate that the greater susceptibility of male to helminth infection may be related to the inhibitory action of androgens on ILC2 biology as mentioned below (50, 51). However, as testosterone can also be converted to estrogens *in vivo* the final demonstration that the androgen-signaling protects from helminth infection through direct signaling in ILC2 still needs to be firmly established.

## Regulation of ILC2 by AR Signaling

As previously mentioned, there is a switch in asthma incidence between sexes at adolescence. Contrary to what happens during childhood, after puberty, women become more susceptible to asthma than men. Since ILC2s play a central role in the induction of airway inflammation, the impact of sex dependent factors on ILC2 biology has been recently investigated (50, 51). ILC2 isolated from the lung of saline- or OVA-sensitized BALB/c mice were expanded with IL-2 and then stimulated with IL-33. Regardless of whether the ILC2s were isolated from inflamed or control tissues, female ILC2s produced significantly more IL-5 and IL-13 than male cells. Enhanced type 2 cytokine gene expression was also observed in IL-33-stimulated female ILC2s compared with male ILC2s (50). Although this study was the first to report a functional difference in male versus female ILC2s, the role of sex hormones was not addressed.

Sex differences in ILC2 homeostatic numbers and in ILC2-driven airway inflammation have been recently reported (51) and the main conclusions are summarized in Figure 1. In absence of inflammation, male mice had reduced number and frequency of ILC2 in the tissues where they particularly accumulate such as adipose tissue and mucosal barriers. In the lungs, female mice had twice as many ILC2s than male mice. ILC2 phenotype was also different between sexes, with ILC2 from male harboring higher level expression of KLRG1 and IL-33 receptor (ST2), suggesting developmental differences between ILC2 from male and female mice. Indeed, the frequency of ILC2p was higher in females. As a consequence, male mice developed less severe lung inflammation when injected with IL-33, which induces ILC2 expansion and activation. This ILC2 sex bias was exquisitely dependent on the male sexual hormones, androgens. Indeed, ovariectomy and ERα-deficiency had no effect on ILC2 development and effector functions in females, ruling out any possible role for estrogens. In favor of a unique role for the male sex hormone androgen in the regulation of ILC2-dependent responses, orchiectomy or AR-deficiency in hematopoietic cells, abolished all phenotypic changes in IL-33-mediated lung inflammation. ILC2p in the bone marrow showed selective expression of the *Ar* gene, while genes coding for the ERs *Esr-1* and *Esr-2* were poorly expressed. This observation is in agreement with the observation that *Ar* is a prototypic ILC2 signature gene in tissue-resident ILC2s (52). Orthologous genes for *Ar* and other ILC2 signature genes have been identified in the most basal vertebrates, suggesting that androgen-signaling might have been selected very early in ILC2 during evolution (53). ILC2p can efficiently replenish the ILC2 compartment following adoptive transfer and thus represent a useful model for tracking ILC2 differentiation and responsiveness to stimuli *in vivo* and *in vitro* (10). The impact of androgen agonists (5α-DHT) and antagonists (Flutamide) on ILC2 differentiation from ILC2p was also examined in this study (51). Whereas androgen inhibited significantly the development of ILC2, AR antagonist alleviates this inhibitory effect, demonstrating that androgen-signaling can regulate the development of the earliest precursors into ILC2. However, as very few ILC2 develop from bone marrow progenitors in adult mice (54), we believe that most of the sex differences observed in the IL-33-driven inflammation model are already imprinted by the preexisting sex bias in tissue-resident ILC2 reflected in the steady-state numbers. Of note, an increased frequency of Ki67^+^ cells among ILC2p and lung ILC2 was observed in female as compared to male mice, suggesting a major effect of androgen on the regulation of ILC2 numbers at steady state, rather than a direct anti-proliferative effect during inflammation (51). However, whether *in vivo* androgen-signaling in tissue-resident ILC2 can protect from IL-33-driven lung inflammation remains to be investigated (Figure 1).

Altogether, these two studies indicate that male sex can not only negatively regulate the functional response of mature ILC2 in a qualitative manner (50) but also the tissue-resident ILC2 numbers at steady state (51). Ligand-induced activation of AR within ILC2 was the main signaling pathway responsible for the sex differences in IL-33-mediated lung inflammation *in vivo* (51). Whether androgen-signaling might also contribute to the negative regulation of ILC2 effector function (50) remains to be carefully addressed.

## Concluding Remarks

Change in gender prevalence from early childhood to adolescence has been observed for many allergic disorders. Although sex steroid hormone estrogens have long been suspected to account for this switch from male to female predominance, so far little evidence exists supporting a disease promoting effect of estrogens through the ER expression in specific immune cell subsets implicated in type 2 inflammation. Focusing on a recently identified subsets of innate lymphoid cells, recent studies pointed to a major impact of sex-linked factors on ILC2 responsiveness (50), and homeostatic regulation *in vivo* (51). The sex differences in ILC2 biology was attributed to androgens that were found to negatively controlled ILC2 development and expansion through AR-signaling (51). Those results, which need to be confirmed in human, may contribute to explain the reversal of the sex ratio for asthma prevalence after puberty. Altogether, this study uncovers a previously unappreciated aspect of ILC2 biology that has important therapeutic implications, particularly in asthma and allergy.

## Author Contributions

J-CG conceived and wrote the review with the help of the other two co-authors.

## Conflict of Interest Statement

The authors declare that the research was conducted in the absence of any commercial or financial relationships that could be construed as a potential conflict of interest. The reviewer, DD, declared a shared affiliation, with no collaboration, with the authors.
